# Insights into recurring multi-country outbreaks of *Salmonella* Strathcona associated with tomatoes, Europe, 2011 to 2024

**DOI:** 10.2807/1560-7917.ES.2025.30.41.2500224

**Published:** 2025-10-16

**Authors:** Vivien Brait, Lena Böff, Natalia Marta Zmarlak-Feher, Nathalie Jourdan-Da Silva, Sara Mazzilli, Maria Pardos de la Gandara, Alexandra Moura, Joel Mossong, Corinna Ernst, Catherine Ragimbeau, Roan Pijnacker, Maren Lanzl, Lin T Brandal, Heidi Lange, Roger Stephan, Michael Biggel, Michelle Raess, Ondřej Daniel, Michaela Spačková, Christina Clarke, Martin Cormican, Aoife Colgan, Patricia Garvey, Paul Mckeown, Rikard Dryselius, Nadja Karamehmedovic, Eva Grilc, Marija Trkov, Mateja Pirš, Derek Brown, Lynda Browning, Ann Hoban, Gauri Godbole, Anais Painset, Marie Anne Chattaway, Anni Vainio, Ruska Rimhanen-Finne, Jennie Fischer, Marina C Lamparter, Wesley Mattheus, Florian Commans, Ana Gverić Grginić, Ivan Mlinarić, Iva Pem-Novosel, Sanja Kurečić Filipović, Ivana Ferencak, Dragan Jurić, Taina Niskanen, Cecilia Jernberg, Valentina Rizzi, Eleonora Sarno, Christian Kornschober, Andreas Wolfsbauer, Dirk Werber, Sandra Simon, Pernille Gymoese, Steen Ethelberg, Luise Müller, Sabine Maritschnik, Anika Meinen, Michael Pietsch

**Affiliations:** 1 Institute for Infectious Disease Epidemiology, Austrian Agency for Health and Food Safety, Vienna, Austria; 2 ECDC Fellowship Programme, Field Epidemiology path (EPIET), European Centre for Disease Prevention and Control (ECDC), Stockholm, Sweden; 3 Department of Infectious Disease Epidemiology, Robert Koch Institute, Berlin, Germany; 4 Postgraduate Training for Applied Epidemiology, Department for Infectious Disease Epidemiology, Robert Koch Institute, Berlin, Germany; 5 ECDC Fellowship Programme, Public Health Microbiology (EUPHEM), European Centre for Disease Prevention and Control (ECDC), Stockholm, Sweden; 6 Mycotic and Parasitic Agents and Mycobacteria, Robert Koch Institute, Berlin, Germany; 7 Santé publique France, Saint Maurice, France; 8 Institut Pasteur, Université Paris Cité, Unité des Bactéries pathogènes entériques, Centre National de Référence des Escherichia coli, Shigella et Salmonella, Paris, France; 9 Health Inspection, Luxembourg Health Directorate, Luxembourg; 10 Laboratoire National de Santé, Department of Microbiology, Dudelange, Luxembourg; 11 Centre for Infectious Disease Control, National Institute for Public Health and the Environment, Bilthoven, Netherlands; 12 Department of Infection Control and Preparedness, Norwegian Institute of Public Health, Oslo, Norway; 13 Institute for Food Safety and Hygiene, National Reference Laboratory for Enteropathogens and L. monocytogenes, Vetsuisse Faculty, University of Zurich, Zurich, Switzerland; 14 Swiss Federal Office of Public Health, Bern, Switzerland; 15 Centre for Epidemiology and Microbiology, National Institute of Public Health, Prague, Czechia; 16 Faculty of Medicine, Charles University, Prague, Czechia; 17 Department of Infectious Disease Epidemiology, National Institute of Public Health, Prague, Czechia; 18 Military Faculty of Medicine, University of Defence, Hradec Králové, Czechia; 19 Reference Laboratory Service, Galway University Hospital, Galway, Ireland; 20 School of Medicine, University of Galway, Galway, Ireland; 21 Health Service Executive (HSE): Health Protection Surveillance Centre, Dublin, Ireland; 22 Health Service Executive (HSE) Public Health: National Health Protection Office, Dublin, Ireland; 23 Public Health Agency of Sweden (PHAS), Solna, Sweden; 24 Center for Communicable Diseases, National Institute for Public Health, Ljubljana, Slovenia; 25 Department for Public Health Microbiology Ljubljana, National Laboratory of Health, Environment and Food, Ljubljana, Slovenia; 26 Institute of Microbiology and Immunology, Medical Faculty, University of Ljubljana, Ljubljana, Slovenia; 27 Scottish Microbiology Reference Laboratories, Glasgow, Scotland; 28 Gastrointestinal and Zoonoses, Public Health Scotland, Glasgow, Scotland; 29 Gastrointestinal Infections & Food Safety (One Health), United Kingdom Health Security Agency, London, England; 30 Gastrointestinal Bacteria Reference Unit, United Kingdom Health Security Agency, London, England; 31 Finnish Institute for Health and Welfare, Department of Public Health, Helsinki, Finland; 32 National Reference Laboratory for Salmonella, German Federal Institute for Risk Assessment (BfR), Berlin, Germany; 33 National Reference Centre Salmonella, Sciensano, Brussels, Belgium; 34 Croatian Institute of Public Health, Division for Epidemiology of Communicable Diseases, Zagreb, Croatia; 35 Emerging, Food and Vector Borne Diseases Section, European Centre for Disease Prevention and Control (ECDC), Stockholm, Sweden; 36 Microbiology and Molecular Surveillance Section, European Centre for Disease Prevention and Control (ECDC), Stockholm, Sweden; 37 European Food Safety Authority (EFSA), Parma, Italy; 38 National Reference Laboratory for Salmonella, Austrian Agency for Health and Food Safety, Graz, Austria; 39 Unit for Enteropathogenic Bacteria and Legionella & National Reference Centre for Salmonella and other enteric pathogens, Robert Koch Institute, Wernigerode, Germany; 40 Department of Infectious Disease Epidemiology and Prevention; Statens Serum Institut, Copenhagen, Denmark; 41 Global Health Section, Department of Public Health, University of Copenhagen, Copenhagen, Denmark; 42 Department of Bacteria, Parasites and Fungi, Statens Serum Institut, Copenhagen, Denmark; 43 Microbiology Service, Croatian Institute of Public Health, Zagreb, Croatia

**Keywords:** foodborne disease, *Salmonella*, disease outbreak, tomatoes, genomics, intersectoral collaboration, multinational aspects

## Abstract

Notifications of *Salmonella* Strathcona infections increased in Europe in 2023 prompting a multi-country outbreak investigation. We aimed to describe the epidemiology of *S*. Strathcona infections in 17 European countries 2011–2024, investigate the genetic relatedness of *S*. Strathcona isolates and identify the vehicle. Cases were persons residing in the study area and with a laboratory-confirmed *S*. Strathcona infection 2011–2024. Confirmed cases had a *S*. Strathcona isolate clustering with the outbreak reference strain in core genome multilocus sequence typing (cgMLST) within 7 allelic differences (AD) and possible cases within 8–13 AD. Probable cases had an epidemiological link to a confirmed case and non-outbreak cases had an isolate > 13 AD from the outbreak reference strain. Since 2011, 662 *S*. Strathcona infections have been identified: 469 confirmed, 161 probable, 13 possible and 19 non-outbreak cases. Median age of the cases was 34 years (IQR: 19–58 years) and 306 (47.5%) were notified in 2023–2024. Most sequenced isolates (469/496; 94.5%) were highly genetically related (≤ 7 AD) over time and across countries, compatible with a common source. Epidemiological and traceback investigations identified small tomatoes from Sicily as the suspect food vehicle. Stringent control measures at the source are needed to stop the contamination and prevent future cases.

**Figure:**
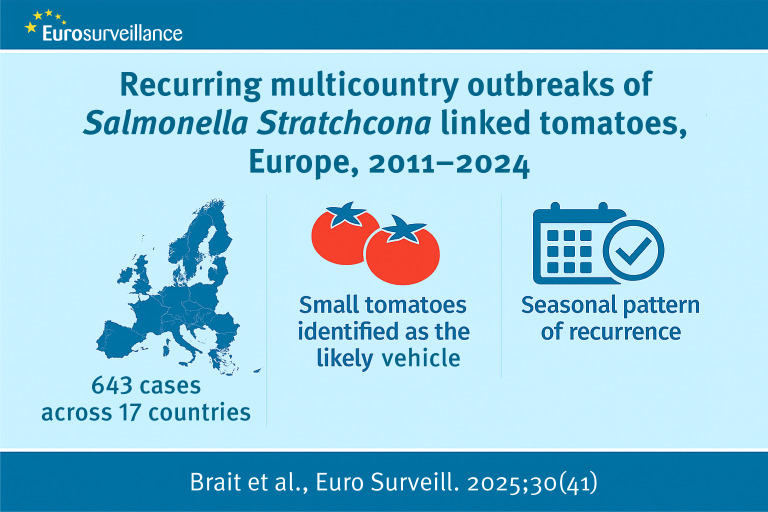


## Introduction

In 2023, non-typhoidal salmonellosis was the second most frequently reported gastrointestinal infection among humans in the European Union and European Economic Area (EU/EEA) countries after campylobacteriosis, accounting for 78,307 cases reported by 30 EU/EEA countries with an incidence of 18.15 cases per 100,000 population [1]. Notification of non-typhoidal salmonellosis is mandatory in 27 EU/EEA countries [1], while in three countries (Belgium, France and the Netherlands), it is voluntary [2]. Non-typhoidal salmonellosis is characterised by diarrhoea, abdominal pain and fever. In some cases, infections may become invasive causing bacteraemia and sepsis, requiring hospitalisation and antimicrobial treatment [3]. The most frequently notified *Salmonella enterica* subspecies *enterica* (*S*.) serovars in humans in the EU/EEA countries are Enteritidis and Typhimurium, including monophasic Typhimurium [2,4].

Prior to 2010, no cases with *S.* Strathcona (antigenic formula: 6,7:l,z_13_,z_28_:1,7) were notified in the EU countries [1]. In early autumn 2011, for the first time, this rare serovar — previously documented only three times globally — was identified in an outbreak affecting multiple European countries, with most cases reported in Denmark. Through consumer purchase data analysis, a case-control study and traceback investigations, the vehicle was found to be small tomatoes originating from Sicily [5,6].

On 27 October 2023, Germany posted a notification on the European surveillance portal for infectious diseases (EpiPulse, maintained by European Centre for Disease Prevention and Control (ECDC)) reporting on 46 cases of *S*. Strathcona occurring since August 2023. Several other EU/EEA countries also reported cases of *S*. Strathcona infections during the same period and subsequently, the outbreak was investigated by the affected countries. A first report on the outbreak was issued in the ECDC Communicable disease threats report on 17 November 2023 [7]. Additionally, retrospective genomic analysis linked the 2011 event with the 2023–2024 outbreak period [4,7].

Given the rarity of the occurrence of *S*. Strathcona before 2011, we suspected that the more frequent occurrence as of 2011 was related to a common source. Therefore, the aims of this study were to: (i) comprehensively describe the epidemiology of *S.* Strathcona infections in the EU/EEA countries, Switzerland, England and Scotland, from 2011 to 2024; (ii) explore the genomic relatedness of strains throughout this period and (iii) to identify the food vehicle for recent *S*. Strathcona outbreaks.

## Methods

### Descriptive epidemiology

Cases with *S*. Strathcona infections were primarily identified through passive routine surveillance systems in the respective countries and supplemented by active case finding during case interviews. During this data collection, all affected countries were invited to collaborate and complete the joint case line list (CLL), which included any *S.* Strathcona outbreak cases identified by national investigations or *S*. Strathcona isolates recognised in the countries from 2011 to 2024. Collaborating countries contributed information about notifying country, sex, age, disease onset date, laboratory receipt date, notification date, sampling date, hospitalisation, clinical outcome, specimen type, travel history, exposure country, whole genome sequencing (WGS) performed and tomato exposure. Additionally, the contributing countries were asked to provide *S*. Strathcona sequences from clinical, environmental, animal or food samples, if available, from 2011 to 2024.

The CLL was the basis to categorise *S*. Strathcona infections. As we suspected a common source and possibly also a common vehicle, we applied a tiered outbreak case definition for the entire study period (Box).

Box **Definitions of cases with *Salmonella* Strathcona infections, Europe, 2011–2024**

**Case:**

• A person residing in an EU/EEA country, Switzerland, England or Scotland with a laboratory-confirmed *S.* Strathcona infection identified 2011–24.

**Outbreak case:**

*Possible outbreak case:*

• *S*. Strathcona isolate without sequencing information or clustering with the outbreak reference strain (SA23–05042; ST2559) within 8–13 AD to the outbreak reference sequence by cgMLST.

*Probable outbreak case:*

• No isolate, but with an epidemiological link to a confirmed outbreak case.

*Confirmed outbreak case:*

• *S*. Strathcona isolate clustering with the outbreak reference strain within 7 AD.

**Non-outbreak case:**

• *S*. Strathcona isolate > 13 AD from the outbreak reference strain.

AD: allelic difference; cgMLST: core genome MLST; EU/EEA: European Union/European Economic Area; MLST multilocus sequence typing; ST: sequence type.

The core genome multilocus sequence typing (cgMLST) thresholds for the different subgroups were selected based on the hypothesis of a common source contamination event before 2011, with subsequent genetic drift over time. Thus, and in order to be in alignment with the European case definition used in the Rapid Outbreak Assessment [4], a cut-off of ≤ 7 AD to the outbreak reference strain (SA23–05042; ST2559) was used for confirmed cases, while 8–13 AD was considered plausible given the estimated *Salmonella* substitution rate of 1–2 single nucleotide polymorphisms (SNPs) per year over the 13-year period [4].

Each of these four subgroups were further broken down into two time periods: the first period from 1 January 2011 to 31 December 2022, and the second period from 1 January 2023 to 31 December 2024.

To better facilitate comparison of case interview information between different countries, an aligned questionnaire was developed, based on national investigation tools from Austria, Czechia, France and Germany, which allowed the pooling of explorative interview results across countries. The time frame for assessing food consumption before the onset of illness varied between national questionnaires, ranging from 3–7 days and up to 2 weeks. The aligned questionnaire covered various food items from relevant food groups, including vegetables, meat, dairy products including cheese, and sweets. Additionally, information on tomato consumption was collected and integrated into the CLL, however, organic production of tomatoes was not consistently specified across all countries.

### Survey for contributing countries

In December 2024, a web-based survey was developed and shared between 17 affected countries to standardise responses on the epidemiological investigations in the countries. The survey included questions on whether *S*. Strathcona outbreak investigations were conducted, including case interviews performed in 2023 and 2024, and earlier if applicable. The survey included closed and open-ended questions to collect information on food exposure periods specific to each country, as well as potential food vehicles identified during the respective outbreak investigations. A table summarising the survey questions can be found in Supplementary Table 4 and the responses in Supplementary Table 1.

### Data analysis

Prior to data processing, the CLL was assessed for the completeness of the reported data variables and the plausibility of exposure location and travel history information. Categorical variables were standardised by recoding inconsistent entries (e.g. for sex, hospitalisation or specimen type) into aligned levels. Variables, such as case definition, were converted to ordered factor levels to ensure consistent reporting across the analysis, and age was transformed to age groups. Descriptive statistics summarised demographic characteristics of the different defined subgroups. For categorical variables, absolute and relative frequencies were calculated, while continuous variables were analysed using means, medians and ranges. To assess incidences across countries, population data from Eurostat [8] and the United Kingdom (UK) Office for National Statistics [9] were employed. Cumulative incidences were calculated for each country as cases per 100,000 inhabitants for 2011–2024. When available, information on food consumption was derived from the aligned questionnaire. Reported consumption was recategorised as ‘Yes‘ for the responses ’Definitely yes‘, ’Probably yes‘, ’Likely yes‘, and as ’No‘ for ’Definitely no‘, ‘Probably no‘ and ’Likely no‘. Food consumption was calculated as relative and absolute frequencies among cases with documented consumption behaviour. When available, the disease onset date was used as the reference date for statistical analyses. If the onset date was unavailable, alternative dates were used as proxies in the following order of priority: sampling date, most recent of either the notification date or the date the specimen arrived at the laboratory.

Data management and statistical analyses were carried out using Microsoft Excel and R version 4.4.1 (https://www.r-project.org). Visualisations were generated with the R packages ggplot2 [10] and incidence [11].

### Binomial probability analysis

In Austria, the probability of obtaining at least the observed number of cases who consumed a particular food during the assumed infection period was calculated. As no official food consumption data were available in Austria, a conservative estimate, i.e. high, was applied. The binomial probability analysis was conducted based on information obtained from the case interviews in 2023. This analysis was used to corroborate the hypothesis, thereby helping to evaluate the need of conducting a case-control study [12].

### Traceback investigations

As a result of findings from case interviews and food purchase records, the Austrian Food Safety Authorities conducted traceback investigations of cherry tomatoes sold by a specific supermarket chain. Based on a Rapid Alert System for Food and Feed (RASFF) notification on 19 January 2024, additional information was obtained on the source of the tomatoes, and which other countries received this product.

### Whole genome sequencing investigations of *Salmonella* Strathcona isolates

The reference sequence corresponds to the first identified case of the seasonal *S.* Strathcona outbreak in Germany in 2023 and was shared internationally as a reference strain via EpiPulse on 27 October 2023. It was submitted to EnteroBase for assigning its HierCC HC5 profile [13]. The WGS and initial data analyses were performed in the respective countries. Collaborating countries provided all *S*. Strathcona isolates which were subsequently analysed at the Robert Koch Institute (RKI) using Ridom SeqSphere software (version 10.0.4) (https://www.ridom.de/seqsphere) [14], including read quality control, de novo assembly (SKESA, version 2.3.0), and cgMLST employing the 3,002 locus *Salmonella* EnteroBase scheme [14,15]. An initial analysis of the sequences submitted to the RKI was conducted, upon which the sequences were categorised and further analysed. Isolates with > 100 missing alleles, or genome coverage < 30 × were excluded.

Core-genome SNP analysis was performed using Snippy version 4.4.5 [16] with N22–0456 as a reference. The reference N22–0456 genome (4.76 Mb, antigenic formula: 6,7:l,z13,z28:1,7) for SNP analysis was constructed through hybrid assembly of long-read (Oxford Nanopore Technologies, Oxford, UK) and short-read (Illumina, San Diego, the United States (US)) data using Unicycler version 0.5.0. Phylogenetic distances were calculated using RAxML version 8.2.9 [17] and visualised with iTOL version 7 [18].

## Results

### Descriptive epidemiology

**Figure 1:**
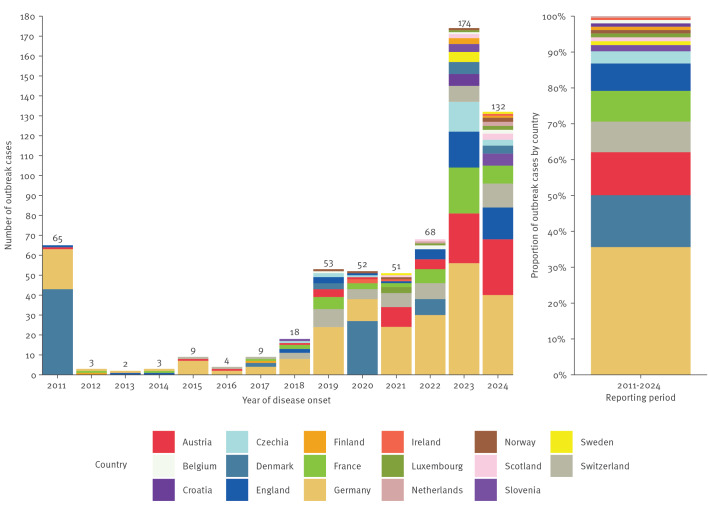
Number of outbreak cases of *Salmonella* Strathcona infection, by year of disease onset, Europe, 2011–2024 (n = 643)^a^

^a^ If the year of disease onset date was unavailable, following dates were used as proxies in the following order of priority: sampling date, most recent of either the notification date or the date the specimen was received in the laboratory.

Between 2011 and 2024, 662 *S.* Strathcona infections were identified in 17 countries: 469 confirmed, 161 probable, 13 possible and 19 non-outbreak cases. Of the 643 outbreak cases, 306 (47.6%) were notified in 2023–2024. The largest number of outbreak cases came from Germany (n = 229; 35.6%), Denmark (n = 93; 14.5%) and Austria (n = 77; 12.0%) (Table 1, Figure 1). The proportion of probable cases of the outbreak cases was lower in 2023–2024 (n = 58; 19.0%) than in 2011–2022 (n = 103; 30.6%).

**Table 1:** Number of cases with *Salmonella* Strathcona infections and sequenced isolates, by country, Europe, 2011–2024 (n = 662)

Characteristics	Confirmed case (n = 469)^a^		Probable case (n = 161)^b^		Possible case (n = 13)^c^		Outbreak case (n = 643)		Non-outbreak case (n = 19)^d^		Sequenced isolates (n = 496)		Total number of cases (n = 662)	
Allelic difference (AD)														
Distance	0–7 AD		NA		8–13 AD		≤ 13 AD or a probable case		> 13 AD		NA			
Time frame	2023–2024	2011–2022	2023–2024	2011–2022	2023–2024	2011–2022	2023–2024	2011–2022	2023–2024	2011–2022	2023–2024	2011–2022	2023–2024	2011–2022
Total number of cases	243	226	58	103	5	8	306	337	2	17	250	246	308	354
Country														
Austria	47	24	6	0	0	0	53	24	0	0	71		77	
Belgium	3	3	0	0	0	0	3	3	0	2	8		8	
Croatia	0	0	6	0	0	0	6	0	0	0	0		6	
Czechia	12	4	4	0	2	0	18	4	0	0	18		22	
Denmark	9	42	0	39	1	2	10	83	0	0	54		93	
England	28	13	4	2	2	0	34	15	1	2	46		52	
Finland	4	0	0	0	0	2	4	2	0	0	4		6	
France	19	9	13	14	0	0	32	23	0	0	28		55	
Germany	85	88	11	44	0	1	96	133	1	13	188		243	
Ireland	1	3	0	0	0	0	1	3	0	0	4		4	
Luxembourg	3	4	0	0	0	0	3	4	0	0	7		7	
The Netherlands	2	1	0	0	0	0	2	1	0	0	3		3	
Norway	2	3	1	0	0	0	3	3	0	0	5		6	
Scotland	0	0	5	0	0	2	5	2	0	0	0		7	
Slovenia	8	1	2	0	0	0	10	1	0	0	9		11	
Sweden	2	0	4	0	0	1	6	1	0	0	2		7	
Switzerland	18	31	2	4	0	0	20	35	0	0	49		55	

AD: allelic difference; cgMLST: core genome multilocus sequence typing; EU/EEA: European Union/European Economic Area; NA: not applicable; ST: sequence type.

^a^ Confirmed outbreak case: person residing in an EU/EEA country, Switzerland, England or Scotland with laboratory-confirmed *S*. Strathcona infection 2011–2024 and with a *S*. Strathcona isolate clustering with the outbreak reference strain (SA23–05042; ST2559) within 7 AD by cgMLST.

^b^ Probable outbreak case: person residing in an EU/EEA country, Switzerland, England or Scotland with a laboratory confirmed infection with *S*. Strathcona or any other *Salmonella* spp. 2011–2024 without a corresponding isolate, but with an epidemiological link to a confirmed outbreak case.

^c^ Possible outbreak case: person residing in an EU/EEA country, Switzerland, England or Scotland with a laboratory-confirmed *S*. Strathcona infection identified 2011–2024 and with a *S*. Strathcona isolate without sequencing information or clustering with the outbreak reference strain within 8–13 AD to the outbreak reference sequence by cgMLST.

^d^ Non-outbreak case: person residing in an EU/EEA country, Switzerland, England or Scotland with a laboratory-confirmed *S*. Strathcona infection identified 2011–2024 and with a *S*. Strathcona isolate > 13 AD from the outbreak reference strain.

**Figure 2:**
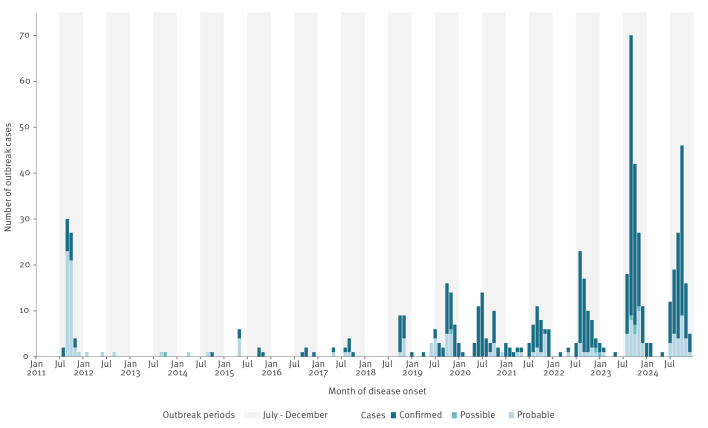
Distribution of *Salmonella* Strathcona outbreak cases, by month of disease onset, Europe, 2011–2024 (n = 643)^a^

^a^ If the year of disease onset date was unavailable, following date were used as proxies in the following order of priority: sampling date, most recent of either the notification date or the date the specimen was received in the laboratory.

Over the past 14 years, a recurring seasonal pattern has been observed, with cases arising from July to the beginning of the following year (Figure 2).

Median age of the cases was 34 years (range: 0–102 years; interquartile range (IQR): 19–58 years), and 55.2% (n = 355) were females (Table 2). The age distribution did not differ between 2011–2022 and 2023–2024. Of the 417 cases with information on hospitalisation available, 154 (36.9%) were hospitalised. The proportion of hospitalised cases decreased from 40.7% (88/216) in 2011–2022 to 32.8% (66/201) in 2023–2024. We had information on specimen type for 489 cases: in 419 (85.7%) of them, *S*. Strathcona was detected in stool samples (Table 2).

**Table 2:** Characteristics of *Salmonella* Strathcona outbreak cases, Europe, 2011–2024 (n = 643)

Characteristics	2011–2022 (n = 337)		2023–2024 (n = 306)		Total (n = 643)	
	n	%	n	%	n	%
Case category						
Confirmed	226	67.1	243	79.4	469	72.9
Probable	103	30.6	58	19.0	161	25.0
Possible	8	2.4	5	1.6	13	2.0
Sex						
Female	185	54.9	170	55.6	355	55.2
Male	145	43.0	136	44.4	281	43.7
Unknown	7	2.1	0	0.0	7	1.1
Age (years)						
0–9	56	16.6	35	11.4	91	14.2
10–19	34	10.1	32	10.5	66	10.3
20–29	49	14.5	53	17.3	102	15.9
30–39	35	10.4	38	12.4	73	11.4
40–49	32	9.5	25	8.2	57	8.9
50–59	35	10.4	24	7.8	59	9.2
60–69	30	8.9	28	9.2	58	9.0
70–79	23	6.8	17	5.6	40	6.2
≥ 80	20	5.9	19	6.2	39	6.1
Unknown	23	6.8	35	11.4	58	9.0
Mean	38		38		38	
Median	34		34		34	
Range	0–97		0–102		0–102	
IQR	17–58		20–56		19–58	
Hospitalisation						
No	128	59.3	135	67.2	263	63.1
Yes	88	40.7	66	32.8	154	36.9
Total	216	64.1	201	65.7	417	64.9
Unknown	121	35.9	105	34.3	226	35.1
Specimen type						
Blood	15	6.8	25	9.3	40	8.2
Faeces	193	87.7	226	84.0	419	85.7
Faeces and blood	4	1.8	0	0	4	0.8
Other	1	0.5	2	0.7	3	0.6
Urine	7	3.2	16	5.9	23	4.7
Total	220	65.3	269	87.9	489	76.0
Unknown	117	34.7	37	12.1	154	24.0
Travel history						
No	163	82.3	127	66.8	290	74.7
Yes	35	17.7	63	33.2	98	25.3
Total	198	58.8	190	62.1	388	60.3
Unknown	139	41.2	116	37.9	255	39.7
Tomato consumption						
No	278	82.5	201	65.7	479	74.5
Yes	59	17.5	105	34.3	164	25.5
Information on detailed food consumption						
No	337	100.0	248	81.0	585	91.0
Yes	0	0.0	58	19.0	58	9.0
Consumption of big tomatoes						
No	14	23.7	39	37.5	53	32.5
Yes	45	76.3	65	62.5	110	67.5
Total	59	17.5	104	34.0	163	25.3
Unknown	278	82.5	202	66.0	480	74.7
Consumption of small tomatoes						
No	10	20.8	25	24.3	35	23.2
Yes	38	79.2	78	75.7	116	76.8
Total	48	14.2	103	33.7	151	23.5
Unknown	289	85.8	203	66.3	492	76.5
Consumption of any tomatoes						
No	3	5.1	13	12.4	16	9.8
Yes	56	94.9	92	87.6	148	90.2
Total	59	17.5	105	34.3	164	25.5
Unknown	278	82.5	201	65.7	479	74.5

IQR: interquartile range.

The highest cumulative incidence across 2011–2024 was observed in Denmark (1.60 cases per 100,000 inhabitants), followed by Luxembourg (1.08/100,000) and Austria (0.87/100,000), as presented in Supplementary Table 2. In 2023–2024, the incidence was highest in Austria (0.58/100,000), Slovenia (0.47/100,000) and Luxembourg (0.45/100,000), as presented in Supplementary Table 2. Among the 388 outbreak cases with known travel history within 7 days before illness, 98 (25.3%) reported travel before illness onset. Of these, 55 had travelled to Italy, 11 to Croatia and 6 to Montenegro. In 2023–2024, more cases had travelled (63/190; 33.2%) than in 2011–2022 (35/198; 17.7%).

### Interview results

From 2011 to 2024, information about tomato consumption was available for 164 (25.5%) outbreak cases from Austria, Czechia, Denmark, France, Germany, Luxembourg, Slovenia, Sweden and Switzerland. Consumption of any tomato type was reported by 148 (90.2%) of 164 interviewed cases (Table 2). Of those with information available, 116 (76.8%) of 151 reported having consumed small tomatoes and 110 (67.5%) of 163 cases having consumed big tomatoes. More detailed information on various food items was available for a subsample of 58 cases from Austria, Denmark, France, Germany, Luxembourg and Switzerland, based on the aligned questionnaire. The five most commonly consumed single food items were potatoes (16/18), eggs (42/54), small panicle tomatoes (42/56), apples (34/49) and cucumbers (37/55). Within this subsample, the pooled consumption rates of any tomato (50/58) and any small tomato (43/57) were among the highest.

Of the 164 interviewed cases, 98 (59.8%) reported likely to have eaten in restaurants, canteens, fast-food places or other places of external food consumption. In 2023, in the national outbreak investigation in Austria, 13 of 14 interviewed cases reported having consumed cherry tomatoes on the vine packed in cardboard tray wrapped in plastic, purchased from a single supermarket chain.

### Binomial probability analysis

The cumulative binomial probability that at least 13 of 14 cases reported the consumption of cherry tomatoes on the vine from the same supermarket chain, even when assuming a (high) background consumption rate of 70% in the general population in Austria, was 0.01, indicating that such a high consumption proportion was unlikely due to chance.

### Survey for contributing countries

Seventeen countries responded to the survey and 12 conducted *S*. Strathcona outbreak investigations in 2023–2024. Case interviews were carried out by the local public health level in one country, while four countries reported conducting interviews at the regional level and seven countries at the national level. Eight countries used a single standardised questionnaire in all case interviews. The time frame for assessing the exposure period of food consumption and dining away from home before the onset of illness varied between national questionnaires, from 3 to 14 days. Prior to 2023, three countries conducted *S*. Strathcona outbreak investigations at national level. Among these, only Denmark had conducted an outbreak investigation in 2011 where Datterino tomatoes were identified as the suspected food vehicle [5].

### Traceback investigations in 2023

During the *S.* Strathcona outbreak in 2023, in Austrian case interviews, organic cherry tomatoes sold in one specific supermarket chain were considered the likely food vehicle. In addition, one patient’s food basket donation containing a batch of organic cherry tomatoes was traced back. This led to a RASFF notification (2024.0384) issued on 19 January 2024, Supplementary Figure 2. In collaboration with the Italian Food Safety Authority through RASFF, the origin of the production and distribution channels for the tomatoes were identified. The implicated batch was likely produced by a certified organic primary production company in Sicily (Italy) and distributed to Austria through an Austrian company. Official inspections at the primary production company in Sicily, along with regulatory water analyses conducted in February 2023 and January 2024, confirmed compliance with hygiene standards and water monitoring for vegetable cultivation. Trace-forward information from the delivery invoices indicated that organic cherry tomatoes from the same organic primary production company in Sicily had been distributed also to further suppliers in Italy, Poland and Slovakia (via a German operator).

### Whole genome sequencing of *Salmonella* Strathcona isolates

**Figure 3:**
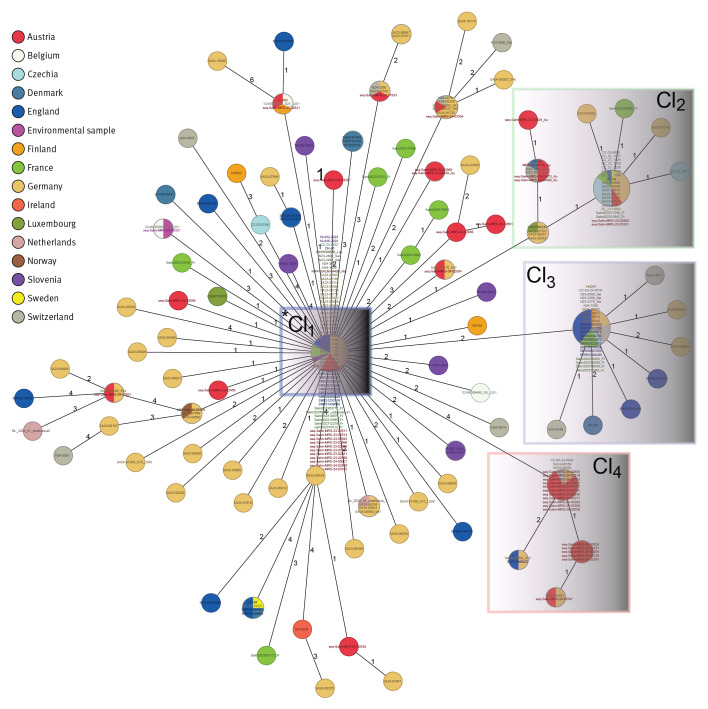
Minimum spanning tree of *Salmonella* Strathcona sequences from confirmed outbreak cases in core genome multilocus sequence typing analysis, by country of isolation, 2023–2024 (n = 243)^a^

AD: allelic difference; cgMLST: core genome multilocus sequence typing; Cl: cluster.

**Figure 4:**
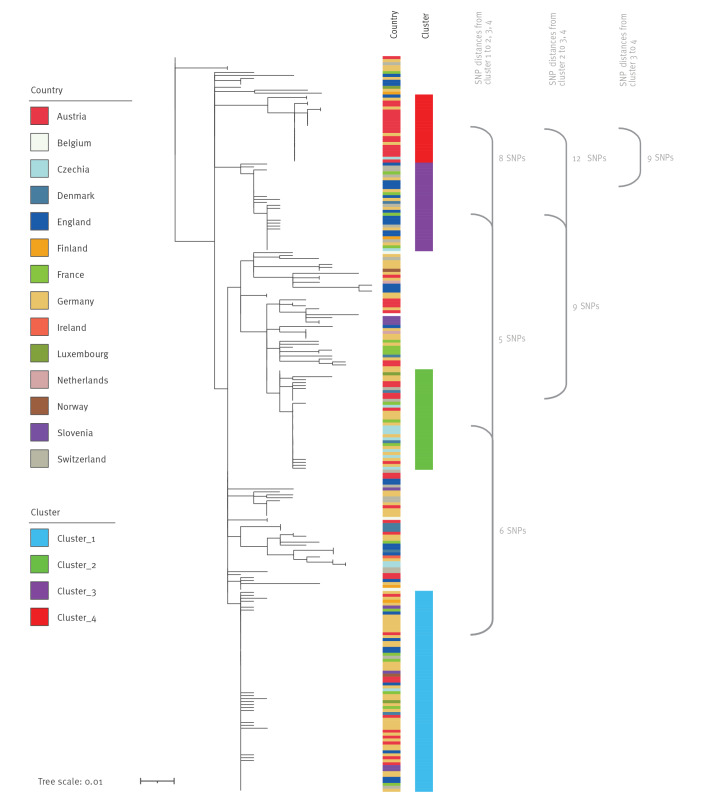
Phylogenetic relationships of *Salmonella* Strathcona sequences from isolates from confirmed outbreak cases, Europe, 2023–2024 (n = 241)^a^

AD: allelic difference; cgMLST: core genome multilocus sequence typing; Cl: cluster; SNP: single nucleotide polymorphism.

In 2011–2024, 496 sequences of clinical isolates and 4 environmental isolates were available from 15 countries. Of these, 469 (94.6%) clinical isolates were within 7 AD difference; this applies to 243 of 250 (97.2%) in 2023–2024 (Table 1). All sequenced isolates from the confirmed cases belonged to the 7-loci MLST type ST2559 and, according to the EnteroBase-derived cgMLST scheme in Ridom SeqSphere, were assigned to CT3910, corresponding to hierarchical cluster HC5_26490 in Enterobase. These 243 isolates formed four large cgMLST clusters (hereby named clusters 1–4), 1–6 AD from the reference sequence, located in cluster 1. The four clusters were composed of isolates from different countries (Figure 3). Isolates from the 34 Austrian cases from 2023 were in each of these clusters. In addition, an Austrian sewage water isolate from 2024 displayed 3 AD to the reference sequence (Figure 3). To achieve higher resolution on the genetic divergence, a whole genome SNP analysis was conducted. This identified four dominant clades, consistent with the cgMLST clusters (Figure 4).

According to the confirmed case definition, 243 isolates from cases with *S*. Strathcona infection from different European countries clustered within 7 AD, number of AD between the isolates is indicated above the branches; the branch length does not illustrate AD between the isolates) from the reference sequence (Cl 1, blue asterisk). Additionally, one environmental isolate from sewage sludge in Austria, collected in 2024, is included (indicated by the red arrow). Based on cgMLST, four large clusters, composed of at least 20 sequences can be distinguished visually: Cl 1: Cluster 1 (62 isolates); Cl 2: Cluster 2 (35 isolates); Cl 3: Cluster 3 (30 isolates); Cl 4: Cluster 4 (23 isolates).

^a^ Confirmed outbreak case: person residing in an EU/EEA country, Switzerland, England or Scotland with laboratory-confirmed *S*. Strathcona infection 2011–2024 and with a *S*. Strathcona isolate clustering with the outbreak reference strain (SA23–05042; ST2559) within 7 AD by cgMLST.

Maximum-likelihood tree based on sequence variation in the core genome of 241^b^ isolates identified as part of the outbreak based on the cgMLST case definition. The first coloured column indicates the country of isolation and the second column highlights the defined outbreak clusters. The core genome displayed 260 SNPs. Maximum SNP distance was 26.

^a^ Confirmed outbreak case: person residing in an EU/EEA country, Switzerland, England or Scotland with laboratory-confirmed *S*. Strathcona infection 2011–2024 and with a *S*. Strathcona isolate clustering with the outbreak reference strain (SA23–05042; ST2559) within 7 AD by cgMLST.

^b^ Due to insufficient sequence quality, the two Swedish isolates had to be excluded from the analysis.

**Figure 5:**
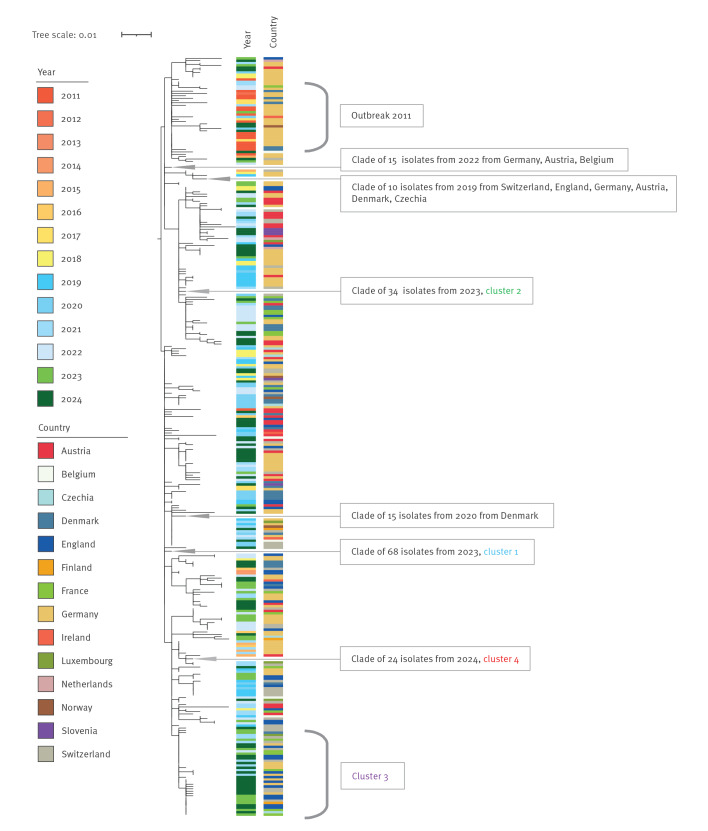
Phylogenetic relationships of *Salmonella* Strathcona sequences from confirmed outbreak cases, Europe, 2011–2024 (n = 467)^a^

AD: allelic difference; cgMLST: core genome multilocus sequence typing; SNP: single nucleotide polymorphism.

Further cgMLST analysis including the 2011–2022 sequences from human and environmental isolates identified 226 (91.9%) of 246 clinical isolates and 3 environmental isolates from sewage sludge from Germany (isolated in 2012, 2018 and 2022) matching the outbreak cluster, as presented in Supplementary Figure 3. Core genome SNP analysis identified the four clusters from 2023 to 2024 across the phylogeny of strains from 2011 to 2024 (Figure 5).

Maximum-likelihood tree based on sequence variation in the core genome of 467^b^ isolates which met the confirmed cgMLST case definition. The first coloured column indicates the year of isolation and the second column indicates the country of isolation. Of 467 isolates, 214 (46%) were found in clades. Six highlighted clades, including outbreak clusters from 2023 to 2024 were collapsed. The core genome displayed 419 SNPs.

^a^ Confirmed outbreak case: person residing in an EU/EEA country, Switzerland, England or Scotland with laboratory-confirmed *S*. Strathcona infection 2011–2024 and with a *S*. Strathcona isolate clustering with the outbreak reference strain (SA23–05042; ST2559) within 7 AD by cgMLST.

^b^ Due to insufficient sequence quality, the two Swedish isolates had to be excluded from the analysis.

Eight additional clinical isolates from 13 possible outbreak cases, from 2011–2022 (n = 3) and 2023–2024 (n = 5), were 8–13 AD from the reference strain in cgMLST analysis. Five possible outbreak case isolates had no sequence information. Additionally, the sequence data also revealed a distinct *S*. Strathcona clade consisting of 19 isolates from non-outbreak cases between 2010 and 2023 from Belgium, Germany and England, which exhibited > 100 AD to the reference sequence, presented in Supplementary Figure 1.

## Outbreak control measures and investigations of tomatoes 2023–2024

After 2023, Austria started intensified testing of imported small tomatoes coordinated by the Austrian National Reference Laboratory for *Salmonella*. In microbiological investigations performed on 77 tomato samples taken between 2023 and 2024 in Austria, Czechia, Germany, Slovenia and Switzerland, *Salmonella* spp. was not detected. In addition, Italian Food Safety Authority announced a national *Salmonella* sampling on cherry tomatoes with focus on production in the region of Sicily.

In 2023, following the traceback investigation of the specific tomato batch in Austria, there were no outbreak control measures implemented in Germany because cherry tomatoes of the implicated lot from this production company were not sold there, nor in Slovakia as no remaining goods were present at the retailer at the time of the inspection.

In January 2024, the Italian Food Safety Authority asked one primary production company in Sicily to perform microbiological testing of the water used for irrigation. *Salmonella* spp. was not detected from the tested samples of irrigation water.

In September 2024, an online intersectoral meeting, including the affected countries, ECDC, European Food Safety Authority (EFSA) and the European Commission (EC) took place to discuss outbreak investigation results, which resulted in the publication of a Joint ECDC-EFSA Rapid Outbreak Assessment [4].

Further precautionary actions were taken in November 2024 when two supermarket chains in Austria voluntarily halted the import of all tomatoes from Italy and withdrew existing stocks from the market. However, no official recalls of tomatoes were issued. Following the halt of imported tomatoes from Italy, only one additional travel-related case from Italy case was notified in Austria.

## Discussion

The results of our multi-country, epidemiological and genomic investigation of *S.* Strathcona infections in Europe from 2011 to 2024 suggest that the vast majority belonged to yearly recurring outbreaks since 2011, caused by a shared common source. Overall, we identified 643 *S.* Strathcona outbreak cases (469 confirmed, 161 probable and 13 possible) across 17 European countries. As not all affected countries joined this collaborative investigation coupled with the fact that case identification was largely based on mandatory notification, the true number of infections is likely substantially higher. Furthermore, the outbreak has continued in 2025 with 29 confirmed outbreak cases from nine countries reported by 4 September 2025 [19]. Given the observed seasonality, more cases are expected to occur this year.

Epidemiological and traceback investigation evidence from Austria identified small cherry tomatoes from Sicily as the suspected food vehicle in 2023. We consider the evidence is strong for several reasons. Firstly, in Austria, case interviews in 2023 (also in 2024, data not shown) revealed a remarkably consistent common consumption pattern of small tomatoes on the vine, including similar preferences for product packaging types and purchasing habits. Our binomial probability analysis suggested that the high consumption frequency was unlikely to be due to chance, and we considered it unlikely that a case-control study would produce a different result. Secondly, in France, cherry tomatoes were among the most reported food items and in Germany, general tomato consumption was the highest single food category reported among cases. Thirdly, in 2024, the Italian Food Safety Authority issued a RASFF notification regarding a national food-borne outbreak of *S*. Strathcona in several schools in Italy, where *S*. Strathcona was detected in a meal consisting of cherry tomatoes and pesto that had been consumed by the patients [4]. Subsequent traceback also pointed towards cherry tomatoes from Sicily as the common suspected food vehicle.

Since 2011, *S*. Strathcona infections in Europe have occurred in recurring seasonal pattern every year, from midsummer to year-end. We deem it likely that tomatoes may have been the vehicle in most if not all the years. Notably, a *S*. Strathcona outbreak investigation conducted in Denmark in 2011 identified small (Datterino) tomatoes from Sicily as the vehicle of the infection. At the time, these tomatoes were not widely distributed in Europe, but had been sold in the other European countries, where cases were reported [6]. This is supported by the fact that Datterino tomatoes are typically in season from early summer to early autumn, with a peak harvest period between July and October [20]. Thus, both the Austrian (2023) and Danish investigations pointed to small tomatoes from Sicily as the suspected vehicle of infection. Furthermore, epidemiological investigations from seven countries in 2024 showed that 90% of interviewed cases had consumed any type of tomato. The consistent seasonal case occurrence and the stable pattern with respect to age and sex throughout the years suggest both a seasonal food product and a consistent population segment with similar food consumption habits over the years. The recurring seasonal pattern suggests that the tomato-growing environment could be a persistent source of contamination across harvest seasons [21].

The genomic analysis revealed high clonality among most clinical isolates from 15 countries from 2011 to 2024, which is compatible with a shared, common source. The selected cgMLST threshold of 7 AD to the reference strain for confirmed cases, while relatively high compared with other *Salmonella* outbreaks [22,23], was chosen to account for the expected genetic drift over the extended time frame (2011–2024).

This threshold may represent a limitation, as it increases the risk of including unrelated cases. However, it was considered appropriate given the observed SNP distances [5-11] between outbreak clades and the hypothesised scenario of a single introduction in or before 2011 followed by persistence and diversification within food production or processing environment. In addition, the comparison between using a threshold of 7 AD vs 5 AD showed that only 40 fewer cases would have been classified as confirmed, suggesting that a more conservative threshold would have led to similar results.

The annual and seasonal reappearance of this serovar in Europe further supports this theory, indicating a possible environmental or agricultural reservoir, such as contaminated water, which facilitates its persistence. Findings of isolates with > 200 AD divergence in Germany and England between 2011 and 2023 suggest that *S*. Strathcona in general is genetically more diverse than previously assumed and this points to the possibility that this serovar has persisted for a considerable period in a specialised niche. It may have potentially gone undetected due to its association with environments or reservoirs that do not commonly result in human disease. These findings support the validity of cgMLST-based approaches for outbreak investigation, while highlighting the importance of interpreting thresholds within the specific epidemiological context.

Fresh tomatoes are an established vehicle for *Salmonella* outbreaks, as exemplified by outbreaks in Sweden [24], Finland [25] and the US [26,27]. This may be related to consumption of tomatoes without cooking [21]. Pathogens may not only adhere to the surface but also internalise within the plant tissues [28]. This indicates washing of tomatoes may not necessarily be an effective preventive measure. Nevertheless, following proper hygiene practices at home, including handwashing, rinsing fresh produce and avoiding cross-contamination remains of importance to prevent illness. Contamination of tomatoes can happen at various points from the farm-to-fork, such as farm, packinghouses, or fresh-cut processing facilities [26]. They can potentially become contaminated with faeces from wild animals [29] or migratory birds [30,31]. Since water used for irrigation does not need to be potable, this may be a further source of contamination. Additionally, during water restrictions, different types of water can be used. Surface waters, such as rivers, streams or lakes, are more susceptible to contamination than protected sources like wells. The sewage sludge isolates from Germany and Austria support the hypothesis that sewage water can carry harmful pathogens and could be transferred to crops when used for irrigation or applied as a fertiliser [21].

Our study has several limitations. Firstly, tomatoes are a frequently consumed food item [32], making it challenging to identify their disproportionately frequent consumption. This is further complicated by the fact that they are often mixed with other ingredients in ready-to-eat foods, and therefore not always remembered by consumers when recalling their food history. A high proportion of cases reported eating in restaurants and other food service establishments, which also makes the recollection of exact ingredients harder to remember. In addition, not all information about food exposure and purchasing behaviour was collected consistently across the collaborating countries and the exposure period before illness onset varied between different investigation teams. Notably, despite thorough interviews, patients did not recall consuming certain food items, highlighting the limitations of self-reported food histories and the value of objective data sources for identifying contaminated food items. These factors may explain why tomato exposure was not consistently identified in national outbreak investigations in the collaborating countries and explain why the comparability across countries is limited. Furthermore, it is plausible that small tomatoes were the vehicle throughout this period. However, since no intensive epidemiological investigations were conducted between 2011 and 2023, this remains speculative. The observed change in the food vehicle from Datterino to cherry tomatoes likely may be due to changes in crop cultivation rather than a change in the underlying source. Using consumer purchase data should be emphasised as a useful tool; it was key to solving the original Danish outbreak in 2011. It was only when consumer purchase data pointed out the specific type of tomatoes that the Datterino tomatoes hypothesis emerged. Furthermore, consumer purchase data were also crucial in the traceback and trace-forward investigation to link to other affected countries. However, not all countries would be able to use consumer data due to data protection issues.

## Conclusion

Our investigations suggest recurring outbreaks in Europe since 2011 with a seasonal pattern of *S*. Strathcona cases that are linked to small tomatoes originating from a common source. The investigation exemplifies the added value but also the need of extensive collaborative cross-border and cross-sectoral investigations, ideally supported by ECDC and EFSA, to address complex food-borne outbreaks. To prevent future *S.* Strathcona infections in Europe, further investigations into the exact source(s) of the contamination with subsequent implementation of targeted measures are necessary.

## Data Availability

Epidemiological data are available upon reasonable request. Genome data were uploaded to European Nucleotide Archive (ENA) and the Sequence Read Archive (SRA) databases, under the accession numbers detailed in Supplementary Table 3.

## Note

Some authors are employed with the European Food Safety Authority (EFSA). However, the present paper is published under the sole responsibility of the authors and may not be considered as an EFSA scientific output. The positions and opinions presented in this paper are those of the authors alone and are not intended to/do not necessarily represent the views/any official position of EFSA. To know about the views or scientific outputs of EFSA, please consult its website under http://www.efsa.europa.eu.

## Supplementary Data

470000 Supplementary Material
