# Supplementary material for: Insights into recurring multi-country outbreaks of Salmonella Strathcona associated with tomatoes, Europe, 2011 to 2024
Source: Euro Surveill. 2025 Oct 16;30(41):2500224. doi: 10.2807/1560-7917.ES.2025.30.41.2500224 (PMC12534777; doi:10.2807/1560-7917.ES.2025.30.41.2500224)
Supplement: Supplementary Material [file 25-00224_MARITSCHNIK_Supplement.pdf]

Supplementary Material for *Salmonella* Strathcona Manuscript (2025)

This supplementary material is hosted by Eurosurveillance as supporting information alongside the article 'Insights into recurring multi-country outbreaks of Salmonella Strathcona associated with tomatoes, Europe, 2011 to 2024', on behalf of the authors, who remain responsible for the accuracy and appropriateness of the content. The same standards for ethics, copyright, attributions and permissions as for the article apply. Supplements are not edited by Eurosurveillance and the journal is not responsible for the maintenance of any links or email addresses provided therein.

Supplementary Figure 1. MST of available *S. Strathcona* human and environmental isolates, coloured by their allelic distance from the reference sequence. A total of 496 human and 4 environmental isolates from 2011-2024 were included in the analysis. A high proportion (95%) of sequences (N=469) matched the *S. Strathcona* confirmed case definition. Eight isolates matched the definition for possible outbreak cases (in yellow) and are still genetically related to the outbreak event. Twenty-one isolates matched the definition for a non-outbreak case and are genetically unrelated *S. Strathcona* events.

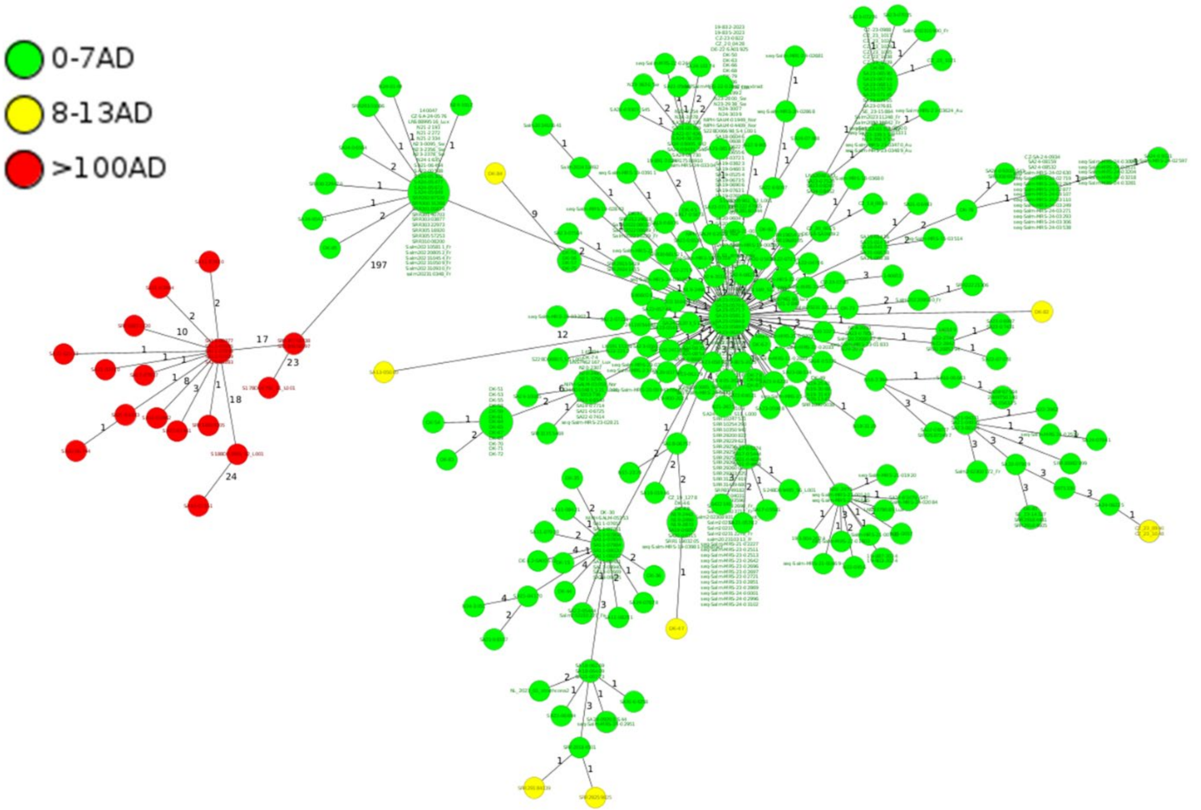

Supplementary Figure 2. Results from traceback investigation to Sicily based on a food basket donation containing organic cherry tomatoes, Austria, 2023

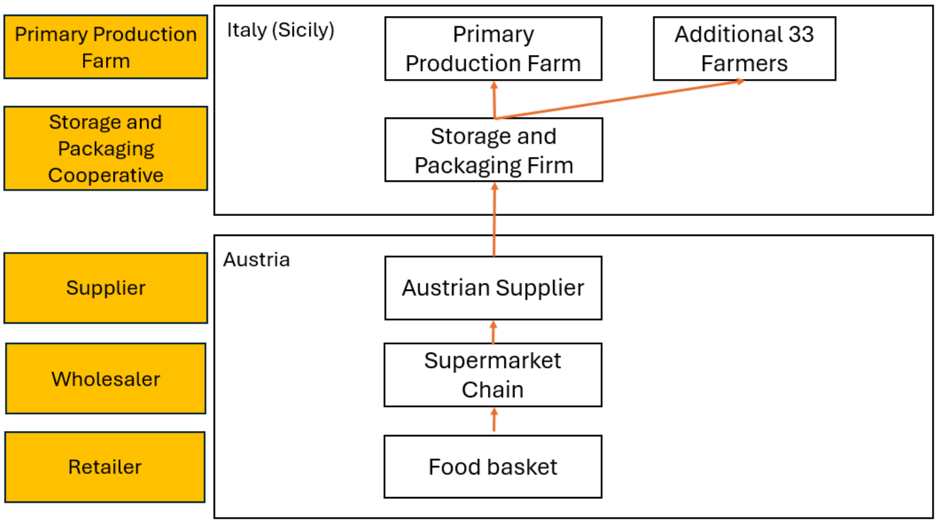

Supplementary Figure 3. MST of historical *S. Strathcona* isolates (2011-2022), coloured by year of isolation. A total of 226 human isolates from confirmed historical cases match the isolates from 2023-2024. Additionally, three environmental samples from German sewage water collected in 2012, 2018, and 2022 are classified within the cluster (indicated by red arrows). Representative 2023-2024 isolates from clusters Cl 1, Cl 2, Cl 3, and Cl 4 are colour-coded in green. The reference sequence from Cl 1 is located in the central sequence group (black, dashed bracket).

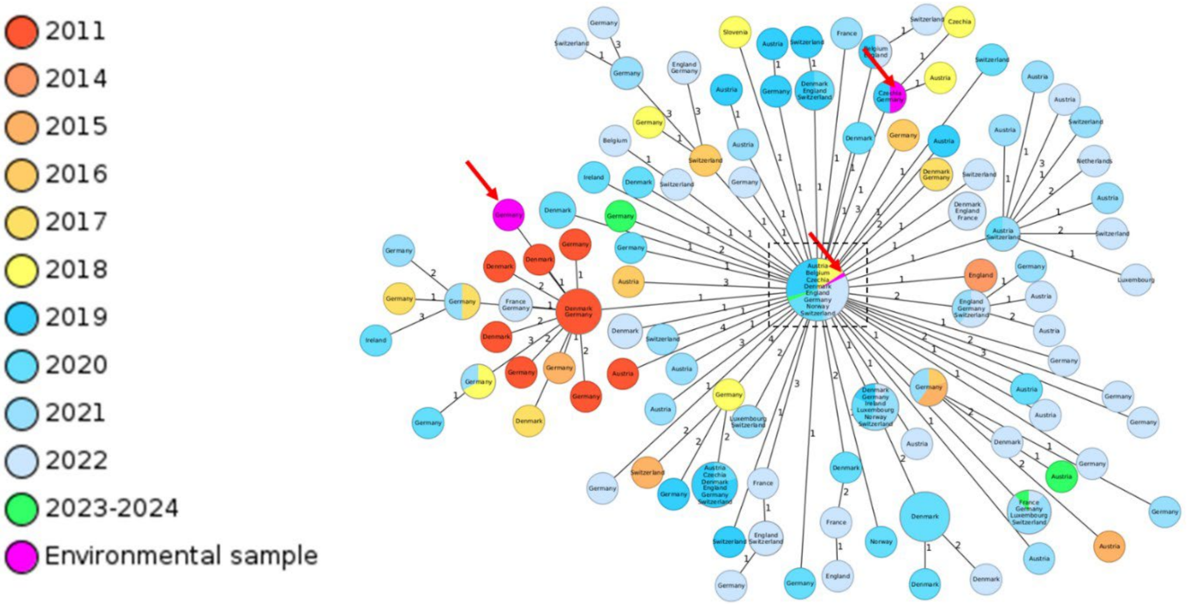

Supplementary Table 1. Results of the web-based survey on *S.* Strathcona outbreak investigations in contributing countries, 2023-2024 (n=17)

Country	Outbreak Investigation Performed	Level of Investigation	Standardized Questionnaire	Days Before*
Austria	Yes	National level	Yes	7
Belgium	No			
Croatia	No			
Czechia	Yes	Regional level	No	3
Denmark	Yes	National level	Yes	7
England	Yes	National level	No	Varied
Finland	No			
France	Yes	National level	Yes	7
Germany	Yes	National level	Yes	3
Ireland	Yes	Regional level	Yes	3
Luxembourg	Yes	National level	Yes	7
Netherlands	Yes	National level	No	
Norway	No			
Scotland	Yes	Local level	No	Varied
Slovenia	Yes	Regional level	Yes	3
Sweden	Yes	Regional level	Yes	7
Switzerland	No			

* Days before = Food history before disease onset

Supplementary Table 2: Cumulative incidences of *S*. Strathcona cases per 100,000 population in EU/EEA countries, Switzerland, England and Scotland, 2011-2024, stratified by outbreak period

Country	Cummulative incidences		
	2011-2022	2223-2024	Total period
Austria	0.27	0.58	0.87
Belgium	0.03	0.03	0.05
Croatia		0.16	0.16
Czechia	0.04	0.17	0.21
Denmark	1.44	0.17	1.60
England	0.03	0.06	0.08
Finland	0.04	0.07	0.11
France	0.03	0.05	0.08
Germany	0.16	0.11	0.28
Ireland	0.06	0.02	0.08
Luxembourg	0.62	0.45	1.08
Netherlands	0.01	0.01	0.02
Norway	0.06	0.05	0.11
Scotland	0.04	0.09	0.13
Slovenia	0.05	0.47	0.52
Sweden	0.01	0.06	0.07
Switzerland	0.41	0.23	0.64

Supplementary Table 3: Overview of S. Strathcona human isolate sequences available in the study. The number of sequences per country is grouped by period (recent vs. historical). Classification into "confirmed," "possible," and "not outbreak" is based on the case definition. The columns labelled "cgMLST" and "SNP" indicate which sequences were used in each respective analysis presented in the study.

Country	Sequences (2023-2024)					Sequences historical (2011-2022)					Total available sequences
	confirmed	possible	not outbreak	cgMLST (Fig 3)	SNP (Fig 4)	confirmed	possible	not outbreak	cgMLST (Fig 5)	SNP (Fig 6)	cgMLST all (Suppl Fig 1)
Austria	47			47	47	24			24	24	71
Belgium	3			3	3	3		2	3	3	8
Croatia											0
Czechia	12	2		12	12	4			4	4	18
Denmark	9	1		9	9	42	2		42	42	54
England	28	2	1	28	28	13		2	13	13	46
Finland	4			4	4		2				4
France	19			19	19	9			9	9	28
Germany	85		1	85	85	88	1	13	88	88	188
Ireland	1			1	1	3			3	3	4
Luxembourg	3			3	3	4			4	4	7
Netherlands	2			2	2	1			1	1	3
Norway	2			2	2	3			3	3	5
Scotland							2				0
Slovenia	8			8	8	1			1	1	9
Sweden	2			2	[a]		1				2
Switzerland	18			18	18	31			31	31	49
TOTALS	243	5	2	243	241	226	3	17	226	226	496[b]

[a]Torrent sequences, not used for SNP analysis

[b]cgMLST analysis of all available sequences contained confirmed, possible and non-outbreak isolates from the recent and historical period in each country.

Supplementary Table 4: Survey instrument used to collect information from countries regarding their national investigations of the *Salmonella* Strathcona outbreak in 2023 and 2024, including case interview practices, levels of public health involvement, use of standardized questionnaires, and historical outbreak data.

Nr.	Survey Question	Response Options / Instructions
1	Contributing country	[Text field]
2	Please write your affiliations in the field below	[Text field]
3	Did your country conduct a national S. Strathcona outbreak investigation in 2023 including case interviews?	☐ Yes ☐ No
4	Who conducted S. Strathcona case interviews (food trawling questionnaires) in 2023?	☐ Local level only ☐ Regional level only ☐ National level only ☐ A combination of public health levels
5	Did your country conduct a national S. Strathcona outbreak investigation in 2024 including case interviews?	☐ Yes ☐ No
6	Who conducted S. Strathcona case interviews (food trawling questionnaires) in 2024?	☐ Local level only ☐ Regional level only ☐ National level only ☐ A combination of public health levels
7	Were S. Strathcona interviews conducted using a standardized questionnaire?	☐ Yes ☐ No
8	How many days before disease onset were cases queried on their food history?	[Please indicate number of days, e.g., 3 days, 7 days, or varied]
9	Were national S. Strathcona outbreak investigations conducted in your country before 2023?	☐ Yes ☐ No
10	If yes, please provide details on similar S. Strathcona investigations, including year(s) of the outbreak(s), number of cases reported, food items implicated (if any), any common sources identified across outbreaks	[Open text field – no word limit]
